# The Role of Public Health Interventions and Control Measures During Mpox Outbreaks: Enhancing Strategies for Disease Prevention and Management

**DOI:** 10.1002/puh2.70103

**Published:** 2025-08-11

**Authors:** Muhammad Shaheer Bin Faheem, Ahmed Ali Khan, Qasra Faheem, Hafiza Qurat Ul Ain, Wajiha Imam, Yusra Aimen, Raheel Ahmed

**Affiliations:** ^1^ Department of Internal Medicine Karachi Institute of Medical Sciences (KIMS) Karachi Pakistan; ^2^ Foundation University Medical College Islamabad Pakistan; ^3^ CMH Multan Institute of Medical Sciences Multan Pakistan; ^4^ Imperial College London London UK

**Keywords:** COVID‐19, GeneXpert, lymphadenopathy, monkeypox, nucleic acid amplification tests

## Abstract

Mpox (formerly—monkeypox) is the second viral pit break after COVID‐19. It is a zoonotic viral illness caused by an *orthopox virus* belonging to the same genus as various cowpox and vaccinia viruses. The mpox virus has two clades, clade IIb, responsible for the disease's global expansion in 2022. The virus can be isolated from rodents, squirrels, and dormice; however, the actual reservoir is unknown. Animal‐to‐human and human‐to‐human transmission can occur through noninvasive as well as invasive routes. The disease is more prevalent among homosexuals and injectable drug users, as long‐term contact is required for transmission. The disease typically presents with fever, myalgia, rash, and lymphadenopathy following an incubation period of 1–2 weeks. Skin lesions are considered preferred diagnostic specimen, with polymerase chain reaction (PCR) remaining the gold standard for confirming diagnosis. Symptomatic treatment antipyretics, antihistamines, and warm baths are given. Oral and intravenous antivirals like tecovirimat and cidofovir are used only in emergency settings, as the clinical trials on their efficacy are still in progress. Vaccinia intravenous immunoglobulins IVIG can be used in immunocompromised individuals. ACAM 2000, IMVAMUNE, and Dryvax are the available vaccines. Non‐pharmacological interventions like hand hygiene, social distancing, and personal protective equipment can significantly reduce viral transmission, whereas early diagnosis can limit the prevalence by providing timely public health interventions.

## Graphical abstract

This paper highlights key public health interventions and control strategies implemented during mpox outbreaks, emphasizing early detection, targeted prevention, and the role of vaccination and education in mitigating disease transmission.

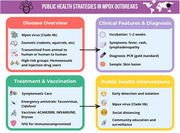

## 1 Mpox Epidemiology, Clades, and Outbreak Patterns

Mpox is considered the current most prevalent outbreak after the COVID‐19 pandemic. It was first discovered in macaque monkeys shipped from Singapore to Copenhagen, Denmark, in 1958, with the first human infection found in a 9‐month‐old infant in Congo. Subclinical disease may arise from indirect or low‐level exposure primarily among individuals residing in or near forest regions [1, 2]. In 2003, mpox virus infection was reported in the United States, marking its spread outside Africa due to animal‐to‐human interaction. Between 2018 and 2021, cases of mpox virus were observed in the United States, Singapore, the United Kingdom, and Israel, whereas the virus had spread to 31 countries by 2022, including cases with no travel history to endemic regions [3]. According to the most recent data from the Centers for Disease Control and Prevention (CDC), over 100,000 cases have been reported across 122 countries from the initiation of disease surveillance in 2022 till July 2024 [4].

**FIGURE 1:**
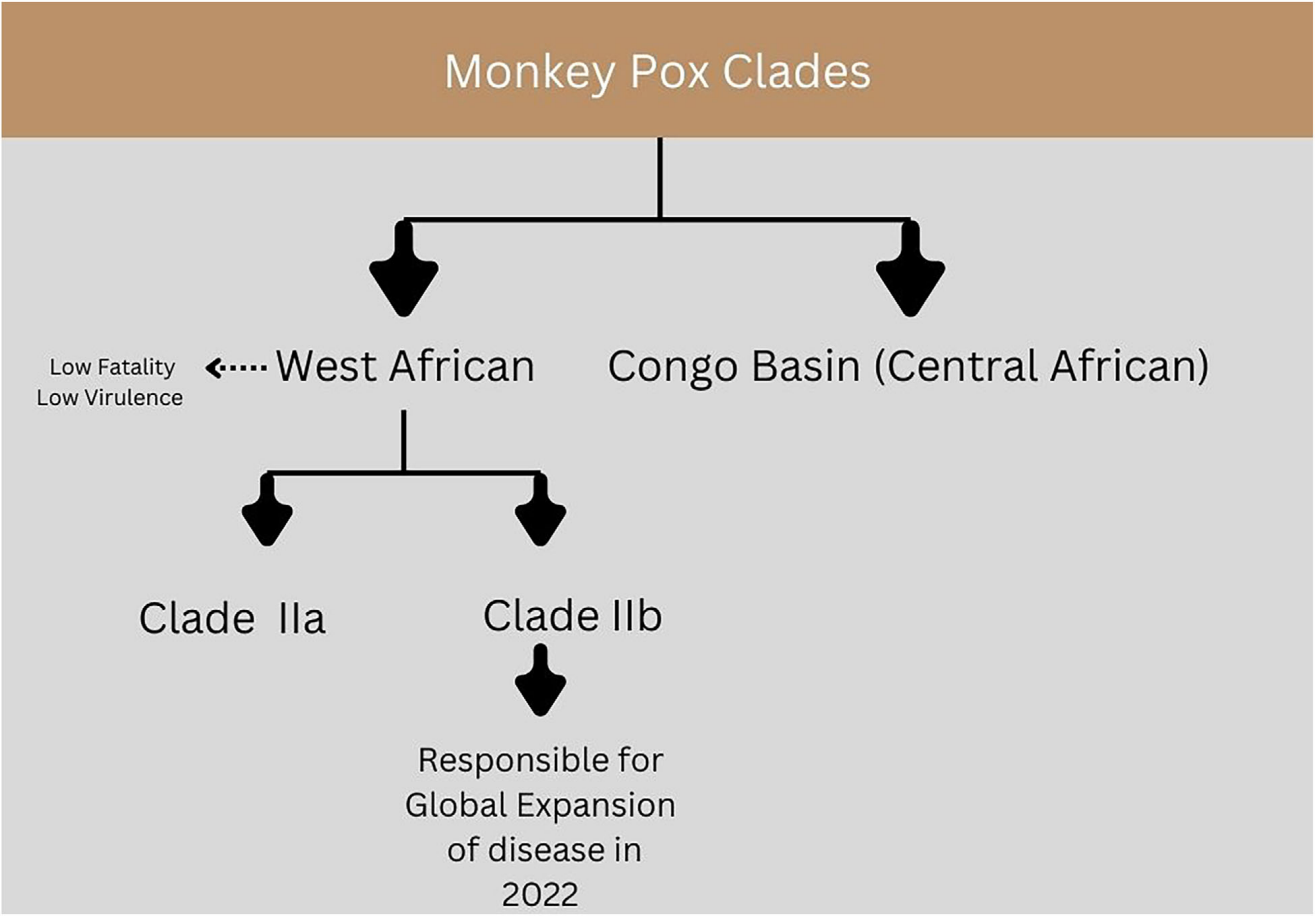
Mpox clades.

Mpox is a zoonotic viral illness caused by an *orthopox virus* belonging to the same genus as variola, vaccinia, and cowpox viruses, resulting in a rash somewhat similar to but milder than smallpox. Mpox is a double‐stranded DNA virus with a large genome comprising almost 200 kb pairs encoding 190 proteins to assemble viral particles that infect and interfere with host processes [5]. The mpox virus has two clades: clade I (with subclades Ia and Ib) and clade II (with subclades IIa and IIb), as shown in Figure 1. Clade I is the strain responsible for causing disease in the Congo Basin or Central Africa and has a 1%–12% fatality rate. The Congo basin continues to experience routine outbreaks of clades Ia and Ib, with the recent spread of clade Ib beyond Africa as of August 2024 [6]. In comparison, clade II is responsible for causing disease in West Africa and has a relatively lower virulence and fatality rate (0.1%) [3, 5]. Clade IIb in particular was responsible for the massive outbreak in 2022 and the global expansion of the disease that continues to date [7, 8].

## 2 Transmission and Reservoirs of Mpox

Although the exact reservoir of this virus is unknown, it has been isolated from rodents and non‐primate animals like squirrels, dormice, and monkeys in Africa. Humans and animals are incidental hosts for this virus. Animal‐to‐human transmission can occur through noninvasive routes via infected animals like petting, touching, and cleaning cages, whereas human‐to‐human transmission manifests through respiratory droplets, direct contact with infected sores facilitated by a breach in the skin or mucosa, vertical transmission to the fetus leading to congenital mpox, and percutaneous transmission after needle stick injuries from infected needles [5]. The majority of the populations in the 2022 outbreak were homosexuals, bisexual men, or those involved in group sex or recreational drugs, highlighting a long‐term contact in transmission [5, 9]. Consumption of partially cooked food, eating bushmeat, or wild game might be sources of infection. Furthermore, mpox virus DNA has been detected in excreta samples [3].

## 3 Clinical Course and Clade‐Based Differences in Mpox Presentation

**FIGURE 2:**
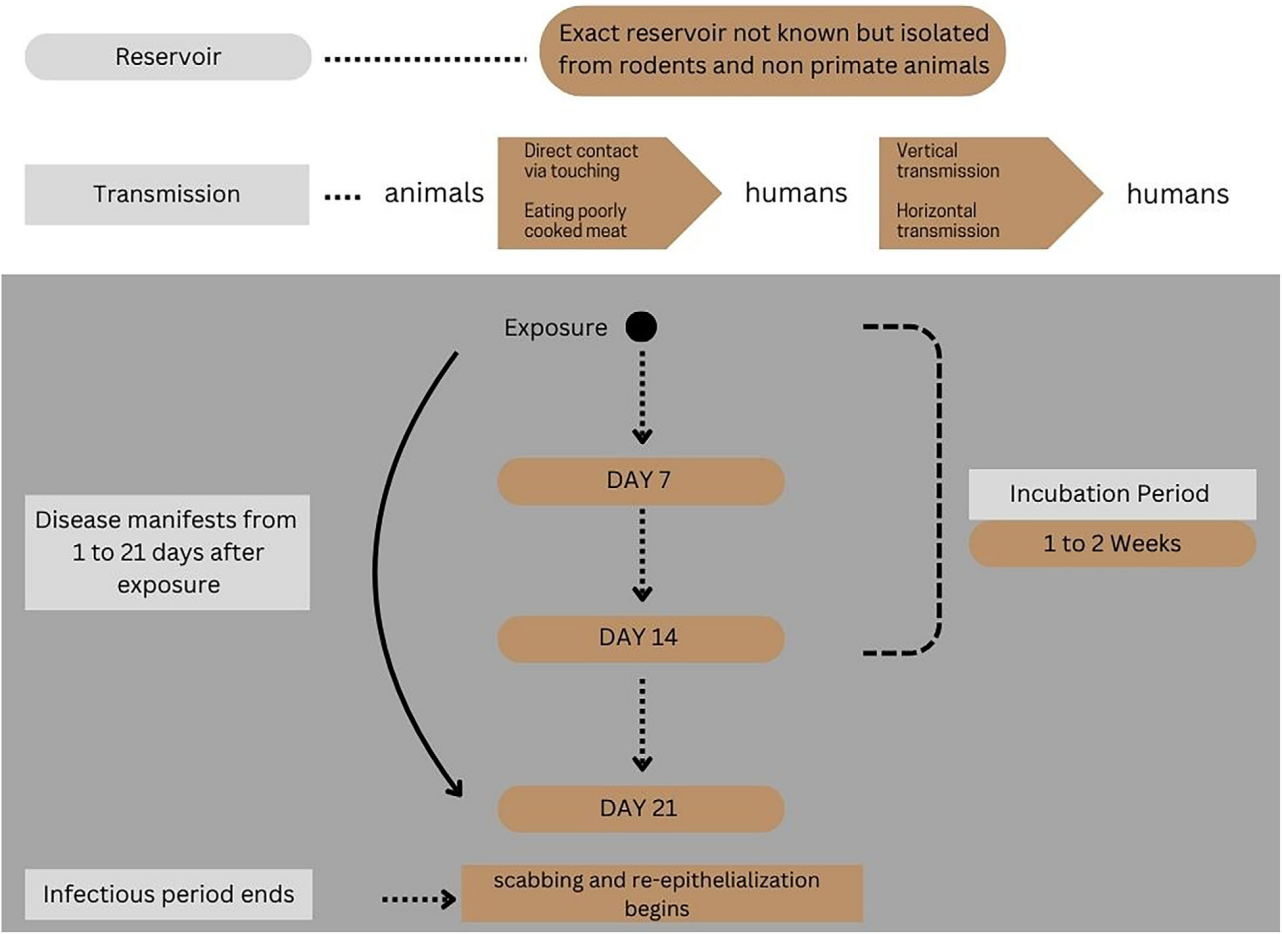
Mpox life cycle.

The clinical course and disease manifestations are notably different between clades I and II. The incubation period of mpox typically ranges from 4 to 21 days with earlier onset of symptoms observed among patients with history of animal bite or scratches [10]. However, the clades responsible for recent outbreaks showed shorter incubation periods ranging from just 7 to 10 days [11]. Figure 2 depicts the lifecycle and clinical course of the mpox virus. Mpox symptomatology is characterized by an initial prodromal phase, in which the patient complains of systemic symptoms like headache, fever, sore throat, myalgias, fatigue, and lymphadenopathy [12]. Lymphadenopathy is the distinguishing feature between mpox and smallpox [3]. Systemic symptoms (fever, headache, and myalgias) and generalized lymphadenopathy were seldom seen as a part of the prodromal phase in the 2022 outbreak; some patients even presented with rash without history of systemic symptoms [13]. The prodromal phase is followed by the clinical phase where patients develop a characteristic rash that lasts 1–2 weeks. The rash may begin 1–2 days before or 3–4 days after the onset of the systemic symptoms and starts as macular lesions that evolve into papules, vesicles, and pseudo‐pustules before crusting over, drying, and eventually falling off at the end of 2 weeks [10, 14]. The infectious period starts from the onset of clinical phase until all skin lesions are scabbed, and reepithelialization has occurred [5]. The location of rash helps in distinguishing between the two mpox clades. Early outbreaks by clades Ia and IIa show a generalized rash distribution, whereas in the 2022 outbreak (clade IIb), the rash was localized mainly to the anogenital, oral, and perioral regions. Clade Ib may present with both localized and generalized rashes [15].

## 4 Clinical and Laboratory Diagnostic Tools for Mpox

Mpox is suspected among patients with rash and lesions in the mouth, conjunctiva, vagina, and anorectal region, and skin lesion material swabs, exudates, or vigorous swabbing of skin crusts are recognized as potential specimens [5]. Nucleic acid amplification tests (NAATs) are recommended for viral detection and characterization due to their accuracy, increased sensitivity, and specificity [16]. The quantitative real‐time polymerase chain reaction (PCR) has been endorsed by the World Health Organization (WHO) as the gold standard for mpox viral diagnosis. This method can correctly identify the virus and measure viral load and can also be used alone or in combination with sequencing. Other diagnostic methods include multiplex real‐time PCR techniques to detect multiple targets like mpox and varicella‐zoster virus simultaneously, pan‐orthodox PCR/ESI–MS to identify all orthodox viruses in a single reaction, and loop‐mediated isothermal amplification to differentiate among different strains of mpox and recombinase polymerase amplification [17]. GeneXpert, immunohistochemistry, and cell culture techniques may also be utilized, but they have certain limitations [7]. Serological tests like ELISA can detect IgM antibodies in acutely ill patients (4–5 days after rash), or IgG antibodies in paired serum samples collected 21 days apart, with the first sample collected in the first week. These diagnostic methods are crucial for detection and contribute to the control of mpox by providing rapid identification [5, 7].

## 5 Mpox Prevention, Vaccination, and Treatment Options

The first line of management for mpox focuses on prevention of transmission and vaccination [18]. Measures include avoiding direct contact (with skin, saliva, mucous membranes, etc.), close contact with individuals exhibiting rash similar to mpox, and avoiding contact with wild animals in mpox endemic regions [19]. Disinfecting surfaces (with 70% ethanol, or 2% glutaraldehyde, or 0.5% sodium hypochlorite) inactivates the virus [9]. Frequent handwashing, wearing masks, and practicing safe sex (use of condoms) are important in reducing transmission rates. Additionally, healthcare professionals must use personal protective equipment (PPE) when attending wards with mpox patients. In case of contact or suspected contact with mpox patients, self‐monitoring for prodromal symptoms for 21 days must be done [6, 20].

Vaccinations can significantly reduce the rising transmission and infection rates of mpox. The modified Ankara‐Bavarian Nordic (JYNNEOS) and the ACAM2000 are the currently available vaccines for immunization against both smallpox and mpox [21, 22, 23]. The JYNNEOS is a live vaccine containing replication‐deficient smallpox and monkeypox virus strains. It is administered in a series of two doses (0.5 mL each, 1 month apart) and provides protection by stimulating cellular and humoral immune responses against these viruses [24]. According to the findings from a clinical trial and a 2023 cohort study, patients who completed the two‐series doses for JYNNEOS were at a lower risk of developing mpox symptoms [25, 26]. Further, those who received at least one dose of JYNNEOS (2.1%) had significantly lower hospitalization rates as compared to unvaccinated individuals (7.5%) [27]. Common adverse reactions like localized pain, redness, and itching may be experienced by the patient at the injection site [28].

On the other hand, the ACAM2000 is a second‐generation vaccine derived from the formerly used Dryvax. Dryvax was licensed by the FDA in 1931 for immunization against smallpox; however, it posed serious risks to pregnant women and immunocompromised individuals, and it was also linked to myocarditis and cardiomyopathy. Owing to the limitations of Dryvax, the FDA approved ACAM2000 as a replacement for Dryvax in 2007 [29]. ACAM2000 contains a live, replication‐competent, but weakened form of the virus. The vaccine is administered subcutaneously by jabbing the skin multiple times with a bifurcated needle to deliver a dose of 0.5 mL (two doses, 1 month apart). This induces a localized infection at the injection site and stimulates the immune system to produce antibodies against smallpox and mpox. Individuals receiving ACAM2000 might develop flu‐like symptoms and swelling of regional lymph nodes in addition to the injection site reactions as seen in JYNNEOS recipients [30, 31]. Although both ACAM2000 and JYNNEOS have proven to be beneficial in mitigating mpox transmission, ACAM2000 is a less preferred option because of its serious cardiac adverse effects and risks for infants and pregnant individuals [32].

Currently, clinically effective treatment regimens for mpox are not available, and treatment mainly includes symptom control and patient counseling. Antipyretics (acetaminophen or ibuprofen) are recommended for fever; stool softeners like docusate can relieve painful bowel movements associated with proctitis [33]. Additionally, topical creams (containing 5% lidocaine) [34] and warm sitz baths (Epsom salt or baking soda dissolved in warm bath water) [35] have proven to have a soothing effect for painful anogenital sores.

Antiviral medications may only be prescribed in life‐threatening cases as clinical trials investigating the efficacy and safety of antiviral medications in mpox therapy are still limited. Tecovirimat is an antiviral drug which was approved originally by the FDA for smallpox treatment. The findings from the STOMP trial suggested that tecovirimat was safe to use in critical mpox patients; however, the efficacy was limited [36]. Further clade II mpox patients on tecovirimat monotherapy did not show a reduction in time to clinical resolution nor improved pain control [37]. Therefore, tecovirimat may be preferred in critically ill mpox patients, including immunocompromised individuals, pregnant women, or children under 8 years of age [5].

Cidofovir and brincidofovir (oral analogue of cidofovir) are other antiviral agents that work by inhibiting viral DNA replication, demonstrating significant reductions in viral replication in the respiratory tract of infected mice while highlighting a strong antiviral activity against mpox clade II strains [38]. However, both drugs are associated with gastrointestinal symptoms, such as nausea, vomiting, and diarrhea. Cidofovir in particular has been frequently linked to nephrotoxicity [39], whereas brincidofovir causes derangement of hepatic enzymes and increased bilirubin levels, suggesting hepatotoxicity [40, 41]. These drugs may be good alternative treatment options in tecovirimat‐resistant cases. Moreover, the FDA has approved and recommended the usage of intravenous vaccinia immunoglobulin (VIGIV) in conjunction with antiviral therapies like tecovirimat, cidofovir, and brincidofovir in immunocompromised patients, providing passive immunity [23, 42, 43]. However, the majority of the studies have been conducted on animal models, which warrants multicentered human trials to explore their therapeutic potential in mpox patients.

## 6 Public Health Strategies for Mpox Prevention and Control

The public health interventions focusing on immunization, targeted testing, treatment, and dedicated community education campaigns are found essential in mitigating the infection rates among the high‐risk populations, such as homosexuals and sex workers, and promoting behavioral changes while minimizing virus exposures and transmission [44, 45]. Further existing SARS‐CoV‐2 vaccination frameworks can be utilized for the distribution of vaccines for the accessibility and coverage enhancement [46]. Public health campaigns should educate communities about the benefits of vaccination and the sexually transmitted nature of mpox, which can help reduce stigma and increase uptake [47]. Accurate and prompt diagnosis is crucial in controlling outbreaks, but it is challenging to find a unique PCR‐based diagnostic for mpox due to its resemblance to other *orthopox viruses*, which can lead to false positive results [7]. Additionally, the limited availability of PCR equipment and decreased accessibility, particularly in developing countries, can further impede molecular testing [7]. Therefore, improving diagnostic capabilities and engaging communities in disease surveillance can improve reporting during outbreaks [48, 49]. Improving viral surveillance and response activities requires swift action and global monitoring. It is crucial to emphasize the importance of responding promptly and efficiently to mpox outbreaks. Continuous monitoring of the genetic makeup of this virus is necessary to detect any significant changes, considering the potential for the mpox virus to adapt to the human host. Early detection of cases allows for necessary actions to be implemented to prevent the further spread of the virus. Timely public health interventions and the prompt sharing of surveillance data ensure a coordinated response, with a particular focus on high‐risk areas [7].

## 7 Future Directions and Recommendations

Although enhanced surveillance technologies, community awareness, and global collaborations have significantly improved our ability to monitor and respond to mpox outbreaks, there remains a critical need for extensive research on vaccines. Most of the available efficacy data is based on results from animal model studies, and further research is needed to ascertain the protective effects in humans. Additionally, there is a need to strengthen public health infrastructure and train healthcare professionals to enhance their readiness for future outbreaks.

## 8 Conclusion and Implications for Public Health

In conclusion, a multifaceted approach involving early diagnosis, targeted public health interventions, and community engagement is required for the effective management of mpox outbreaks. By implementing strong surveillance systems, advancing research on vaccines, enhancing capacity building efforts, educating communities, and promoting international collaboration, we can better prepare for and respond to future mpox outbreaks. These strategies not only improve disease prevention and control but also strengthen global public health security, emphasizing the importance of proactive measures in safeguarding public health worldwide.

## Author Contributions

**Muhammad Shaheer Bin Faheem**: conceptualization, visualization, resources, data curation, project administration, writing – original draft, writing – review and editing, validation. **Ahmed Ali Khan**: data curation, resources, methodology, writing – review and editing, writing – original draft. **Qasra Faheem**: investigation, writing – original draft, resources, validation. **Hafiza Qurat Ul Ain**: writing – original draft, visualization, resources. **Wajiha Imam**: writing – original draft, resources. **Yusra Aimen**: validation, investigation. **Raheel Ahmed**: supervision.

## Disclosure

The authors have nothing to report.

## Ethics Statement

The authors have nothing to report.

## Consent

The authors have nothing to report.

## Conflicts of Interest

The authors declare no conflicts of interest.

## Data Availability

No new data generated.
